# 

**Published:** 1887-10

**Authors:** 


### BOOKS AND PAMPHLETS RECEIVED.

BULLETIN OF THE IOWA STATE BOARD OF HEALTH. Des Moines,

BULLETIN OF THE MINNESOTA STATE BOARD OF HEALTH. Redwing.

LETTER ON THE HOG CHOLERA QUESTION, by Frank S. Billings, D. V. M., Director Laboratory, Lincoln, Nebraska.

CATALOGUE AND ANNOUNCEMENT VETERINARY DEPARTMENT UNIVERSITY OF PENNSYLVANIA, 1887–'88. Philadelphia.

ANNUAL ANNOUNCEMENT OF THE ONTARIO VETERINARY COLLEGE, Toronto Canada. Session 1887–'88.

PROSPECTUS ROYAL (Dick) VETERINARY COLLEGE. Edinburgh, 1887–'88.

ANNOUNCEMENT OF GROSS MEDICAL COLLEGE OF DENVER. Session 1887–'88.

ANNUAL REPORT OF THE PRESIDENT OF BROWN UNIVERSITY. Providence, 1887.

COMPARATIVE PSYCHOLOGY. By T. Wesley Mills, M. A., M. D. Reprint from Popular Science Monthly, March, 1887.

EXPERIMENTS ON THE PREVENTIVE INOCULATION OF RATTLESNAKE VENOM. By Henry Sewall, Ph. D., Professor of Physiology in the University of Michigan, Ann Arbor.

ANNUAL REPORT OF THE SPECIAL COMMITTEE ON SURGERY, 1886. Compiled and edited by George Cupples, M. D. From the Texas State Medical Association.

CONTRIBUTIONS TO THE COMPARATIVE CRANIOLOGY OF THE NORTH AMERICAN INDIANS. By R. W. Shufeldt, M. D. Reprint from the Journal of Anatomy and Physiology, Vol. 21.

NOTES ON THE VISCERAL ANATOMY OF CERTAIN AUKS. By R, W. Shufeldt, C. M. Z. S. Reprint, from the proceedings of the Zoological Society of London, 1887.

STUDIES FROM THE BIOLOGICAL LABORATORY. John Hopkins University. Vol, 4, Nos. 1 and 2. N. Murray. Baltimore.

REPORT OF THE ORNITHOLOGIST. C. Hart Merriam, M. D., for the year 1886. Reprint from the Report of the Agricultural Department, Washington, 1887.

PROCEEDINGS OF THE CANADIAN INSTITUTE. Toronto, Vol. IX. No. 2.

VERSLAG VAN DEN TESTAND VAN HET KONINKLIJK ZOOLOGISCHE-BOTANISCH GENOOTSCHAP TE. S.—Gravenhage, over het jaar, 1886.

BULLETIN OF THE AMERICAN MUSEUM OF NATURAL HISTORY. Vol. II. No. 1. May, 1887.

PROCEEDINGS OF THE LINNEAN SOCIETY OF NEW SOUTH WALES. Vol. II. part 1. Sydney, 1887.

